# Dyssynchronous diaphragm contractions impair diaphragm function in mechanically ventilated patients

**DOI:** 10.1186/s13054-024-04894-3

**Published:** 2024-04-02

**Authors:** Benjamin Coiffard, Jose Dianti, Irene Telias, Laurent J. Brochard, Arthur S. Slutsky, Jennifer Beck, Christer Sinderby, Niall D. Ferguson, Ewan C. Goligher

**Affiliations:** 1 Department of Respiratory Medicine, Aix-Marseille University, APHM, Hôpital Nord, Marseille, France; 2 Division of Respirology, Department of Medicine, University Health Network, Toronto, Canada; 3 Interdepartmental Division of Critical Care Medicine, University of Toronto, Toronto, Canada; 4 Keenan Centre for Biomedical Research, Li Ka Shing Knowledge Institute, St. Michael’s Hospital, Toronto, Canada; 5 University of Toronto, Toronto, Canada; 6 Toronto General Hospital Research Institute, 585 University Ave., 9-MaRS-9024, Toronto, ON M5G 2N2 Canada; 7 Department of Physiology, University of Toronto, Toronto, Canada; 8 Institute for Health Policy, Management, and Evaluation, University of Toronto, Toronto, Canada

**Keywords:** Diaphragm dysfunction, Myotrauma, Mechanical ventilation, Acute respiratory failure

## Abstract

**Background:**

Pre-clinical studies suggest that dyssynchronous diaphragm contractions during mechanical ventilation may cause acute diaphragm dysfunction. We aimed to describe the variability in diaphragm contractile loading conditions during mechanical ventilation and to establish whether dyssynchronous diaphragm contractions are associated with the development of impaired diaphragm dysfunction.

**Methods:**

In patients receiving invasive mechanical ventilation for pneumonia, septic shock, acute respiratory distress syndrome, or acute brain injury, airway flow and pressure and diaphragm electrical activity (Edi) were recorded hourly around the clock for up to 7 days. Dyssynchronous post-inspiratory diaphragm loading was defined based on the duration of neural inspiration after expiratory cycling of the ventilator. Diaphragm function was assessed on a daily basis by neuromuscular coupling (NMC, the ratio of transdiaphragmatic pressure to diaphragm electrical activity).

**Results:**

A total of 4508 hourly recordings were collected in 45 patients. Edi was low or absent (≤ 5 µV) in 51% of study hours (median 71 h per patient, interquartile range 39–101 h). Dyssynchronous post-inspiratory loading was present in 13% of study hours (median 7 h per patient, interquartile range 2–22 h). The probability of dyssynchronous post-inspiratory loading was increased with reverse triggering (odds ratio 15, 95% CI 8–35) and premature cycling (odds ratio 8, 95% CI 6–10). The duration and magnitude of dyssynchronous post-inspiratory loading were associated with a progressive decline in diaphragm NMC (*p* < 0.01 for interaction with time).

**Conclusions:**

Dyssynchronous diaphragm contractions may impair diaphragm function during mechanical ventilation.

**Trial registration:**

MYOTRAUMA, ClinicalTrials.gov NCT03108118. Registered 04 April 2017 (retrospectively registered).

**Supplementary Information:**

The online version contains supplementary material available at 10.1186/s13054-024-04894-3.

## Introduction

Mechanical ventilation may cause diaphragm dysfunction by a variety of postulated mechanisms, including overassistance myotrauma, underassistance myotrauma, and eccentric myotrauma [1]. Eccentric myotrauma is thought to occur when the diaphragm contracts while lengthening, resulting in high shear stresses that induce acute injury and weakness. In mechanically ventilated patients, eccentric contractions can occur when the ventilator cycles into the expiratory phase before the neural inspiratory phase is complete, resulting in “post-inspiratory” loading of the muscle. Such post-inspiratory loading occurs during multiple forms of patient-ventilator dyssynchrony [2] and under conditions of expiratory braking, where prolonged diaphragm contractions slow the rate of decrease in lung volume during the early course of expiration to prevent atelectasis [3]. Recent data suggest that post-inspiratory loading may be common in critically ill patients [4].

The hypothesis of eccentric myotrauma as a mechanism of ventilator-induced diaphragm dysfunction is intriguing, but its clinical relevance remains uncertain. Pre-clinical studies have demonstrated that dyssynchronous diaphragm contractions are associated with acute diaphragm injury and dysfunction [5–7] and that eccentric loading results in acute diaphragmatic weakness [8]. Diaphragm biopsies in humans including mechanically ventilated patients manifest evidence of acute load-induced injury and inflammation [9, 10], suggesting that load-induced injury occurs in this setting. It remains unclear however whether dyssynchrony and associated post-inspiratory loading—apart from elevated inspiratory load per se—contribute to the development of diaphragm weakness in the clinical setting. Clinical evidence for eccentric myotrauma would imply that achieving synchrony is critical to diaphragm-protective ventilation.

To characterize in detail the evolution of diaphragm activity over time and to evaluate the influence of inspiratory loading and dyssynchronous post-inspiratory loading on diaphragm function during mechanical ventilation, we conducted a prospective observational cohort study with hourly recordings of diaphragm activity and daily measurements of diaphragm neuromuscular coupling for up to 7 days in mechanically ventilated patients at high risk of requiring prolonged ventilation.

## Methods

### Study population and setting

This prospective physiological cohort study (MYOTRAUMA, ClinicalTrials.gov NCT03108118, date of registration: 04/04/2017) was conducted in three medical-surgical intensive care units in Toronto, Canada. Informed consent was obtained from substitute decision makers prior to enrolment. If no substitute decision maker was available and to facilitate timely evaluation, eligible patients were enrolled by deferred consent and consent for the use of study data was obtained from study participants once they regained capacity. The Research Ethics Boards at University Health Network and Sinai Health System approved the study protocols, and the study was performed in accordance with the ethical standards laid down in the 2008 Declaration of Helsinki. The study is reported in conformance to the STROBE reporting guideline for observational studies [11].

Patients were enrolled if they were intubated for fewer than 36 h for acute brain injury (i.e., stroke or traumatic brain injury), acute respiratory distress syndrome (ARDS), septic shock, or pneumonia. Patients were excluded if they were deemed unlikely to remain on the ventilator for at least 7 days in the judgment of the investigators, if they had received mechanical ventilation for > 48 h in the preceding 6 months, if they were receiving mechanical ventilation for neuromuscular disease or had a high cervical spine injury, if there was a contraindication to esophageal catheterization (e.g., recent upper gastrointestinal surgery, bleeding esophageal varices), or if they had a concomitant acute exacerbation of obstructive airways disease.

### Study measurements

Following enrolment, a nasogastric catheter fitted with esophageal and gastric balloons and a multi-electrode array for monitoring diaphragm electrical activity (Neurovent Research Inc., Toronto, Canada) [12, 13] was placed. Airway pressure (Paw), flow, esophageal pressure (Pes), gastric pressure (Pga), and diaphragm electrical activity (Edi) waveforms were recorded for 5 min on an hourly basis for up to 7 days (or until extubation or death, if earlier) using a dedicated signal acquisition system (Neurovent Research Inc., Toronto, Canada) connected to the ventilator (Servo-i or Servo-U, Getinge, Solna, Sweden). Transdiaphragmatic pressure (Pdi) was computed by real-time digital subtraction of Pes from Pga. Clinical characteristics including age, sex, admitting diagnosis, comorbidities, severity of illness, and organ dysfunction were collected at baseline. Ventilator settings were recorded daily.

To assess changes in diaphragm function over time, diaphragm neuromuscular coupling (computed as ratio of inspiratory swings in Pdi and Edi from onset to peak) was measured once daily in the morning (generally between 8 am and noon) on days when diaphragm activity (Edi > 0 µV) was present (Additional file 1: Figure E1). Neuromuscular coupling (NMC) reflects the overall efficiency of diaphragm muscle performance by normalizing force generation to the level of muscle activation [14–16] in order to account for the effect of volitional effort on force generation (difficult to standardize in critically ill patients with compromised cognition). To minimize the influence of the force–velocity relation of muscle on NMC [17], NMC was measured with the airway occluded to obtain quasi-static conditions [14, 18]. At least 10 intermittent expiratory airway occlusions were applied at random intervals of approximately 60 s. Each occlusion was maintained for the duration of a single neural inspiration (confirmed by the return of Paw and Edi to baseline). Diaphragm thickness (Tdi) and thickening fraction (TFdi) were measured daily by ultrasound according to the previously described technique [19].

### Signal analysis

Recordings were appraised for signal quality independently and in duplicate as detailed in the Supplement; recordings with evidence of substantial artefact in the Edi tracings were excluded from analysis.

Hourly diaphragm activity was computed by averaging the magnitude of each inspiratory swing in Edi from baseline to the peak (∆Edi) from all breaths in the 5-min hourly recording. Pmus was estimated from ∆Edi and airway NMC using the method of Bellani et al. [20], Pmus = NMC * ΔEdi * 3/4. Estimated Pmus was computed for each breath in the recording based on the ΔEdi value for each breath, and then the average value for the recording was taken as the hourly measurement. For this computation, the daily NMC measurement was imputed to the entire 24-h period. In recordings where Edi was absent (i.e., diaphragm inactive), Pmus was taken to be 0 cm H_2_O. Pmus was estimated from Edi rather than Pdi because to our knowledge no similar method for estimating Pdi has been validated.

The presence of dyssynchronous events (reverse triggering, ineffective triggering, breath stacking, premature cycling) and post-inspiratory loading conditions was assessed in each recording by off-line automated signal analysis [21] according to pre-specified event definitions listed in Additional file 1: Table E1. We defined post-inspiratory loading as inspiratory diaphragm activity (based on Edi) occurring during mechanical expiratory conditions (based on flow). Given uncertainty in the exact definition of post-inspiratory loading we pre-specified more conservative (restrictive) and more liberal (sensitive) definitions. Dyssynchronies were classified as present on any given hour if the rate of dyssynchronous events in the hourly recording was ≥ 1 per minute. Post-inspiratory pressure–time product of the diaphragm, a measure of the magnitude of post-inspiratory loading, was estimated from the product of end-inspiratory Edi, the duration of time from the onset of mechanical expiration to the end of neural inspiration (taken as 70% of peak Edi) [22], and the daily measurement of diaphragm NMC.

### Statistical analysis

The original pre-specified sample size of 60 patients (> 300 patient-days) was computed to yield 91% power to detect an interaction between Edi and time on diaphragm thickness, assuming a 2% decrease in diaphragm thickness per day in the absence of diaphragm activity, a 0.1 ± 0.03% increase in the change in diaphragm thickness per day per unit of mean daily Edi (µV), and an average of 5 measurements per patient. After enrolling 49 patients, the study was discontinued in early 2020 due to slow enrolment and the onset of the Covid-19 pandemic. Since we previously reported the association between Edi and time on diaphragm thickness [23], we modified the objectives to focus on the association between post-inspiratory loading and diaphragm NMC.

Continuous variables were described in terms of mean and standard deviation or median and interquartile range, as appropriate for their distribution. Categorical variables were described in terms of proportions. Gaps in time series data for Edi, estimated Pmus, dyssynchrony, and post-inspiratory loading (i.e., data missing subsequent to the first recorded measurement) were imputed using an exponentially weighted moving average procedure; gaps of 6 h or greater were not imputed.

In order to accurately estimate the burden of exposure to different loading conditions during mechanical ventilation, time series data were extrapolated back to time 0 (hour of intubation) by fitting a Bayesian mixed effects cumulative logistic regression model specifying subject-specific intercept and slopes. As a sensitivity analysis, data were extrapolated to time 0 by fitting a spline model to each individual patient’s time series data. Details and rationale for missing data imputation and extrapolation are presented in the Supplement.

The associations of Edi and post-inspiratory loading with the rate of change in NMC over time were evaluated by testing for an interaction between these variables and time. These models were fitted using data from periods with observed or imputed data, but not extrapolated data. All statistical tests were considered significant at approximately *p* < 0.05. Statistical analyses were performed using R version 4.1.2 (www.R-project.org).

## Results

### Study population

Between January 2014 and January 2020, 209 patients were screened and found to be potentially eligible (Additional file 1: Figure E2). Of these, 49 were enrolled, including 5 patients by deferred consent. Two participants enrolled by deferred consent asked to withdraw from study participation upon regaining capacity and were excluded from analysis; two additional patients were excluded from analysis because very few recordings with acceptable quality were obtained, leaving 45 patients for analysis.

Monitoring was initiated within a median of 23 h after intubation (interquartile range 18–33 h, range 4–42 h). The median duration of monitoring in the study was 5 days (IQR 3–7 days). A total of 4508 hourly recordings were collected (median 105 study-hours per participant, IQR 48–141 study-hours per participant). After appraising signal quality, 3669 hourly recordings were accepted for analysis. Data could be imputed for an additional 389 study hours (total 4058 study-hours for analysis). After extrapolating Edi, Pmus, dyssynchrony rates, and post-inspiratory loading from all available data to hours in the first 48 h after intubation when these measurements were not obtained, measurements for analysis were available for 5102 patient-hours. NMC measurements were obtained in 41 patients, allowing Pmus to be estimated for 4918 patient-hours.

Clinical characteristics of study participants at baseline are reported in Table 1. Most participants were intubated for pneumonia or septic shock; a minority were intubated for acute brain injury. One patient was transferred out of the ICU to another hospital before extubation and was lost to follow-up. Thirty-one patients (67%) survived to extubation and ICU discharge. The median duration of ventilation was 5 days (IQR 3–10 days).

**Table 1:** Baseline characteristics

Characteristic	Distribution (mean (SD)) (n = 45)
Age (years)	60 (14)
Sex (female)	24 (53%)
Height (cm)	166 (9)
Weight (kg)	79 (27)
Admitting diagnosis	
Pneumonia	22 (49%)
Acute respiratory distress syndrome	10 (22%)
Non-pulmonary sepsis	7 (16%)
Acute brain injury	6 (13%)
Intracerebral hemorrhage	3 (7%)
Subarachnoid hemorrhage	1 (2%)
Ischemic stroke	1 (2%)
Traumatic brain injury	1 (2%)
Comorbidities	
Asthma	3 (7%)
Chronic obstructive pulmonary disease	5 (11%)
Interstitial lung disease	3 (7%)
Obstructive sleep apnea	5 (11%)
Congestive heart failure	4 (9%)
Cirrhosis	2 (4%)
Chronic kidney disease	5 (11%)
End-stage renal disease on dialysis	4/43 (9%)
Diabetes	8 (22%)
Stroke	2 (4%)
Immunocompromise	5 (10%)
SAPS II score (median, interquartile range)	47 (42–63)
On veno-venous extracorporeal life support	9 (20%)
PaO_2_/FiO_2_ (mm Hg) (median, IQR)	149 (110–170)
Glasgow Coma Scale (median, IQR)	9 (3–15)
Ventilator settings at enrolment	
Mode	
ACVC	5 (11%)
ACPC	33 (73%)
PSV	7 (16%)
Peak pressure (cm H_2_O) (median, IQR)	25 (20–29)
PEEP (cm H_2_O) (median, IQR)	10 (5–12)
Inspiratory pressure (median, IQR)	10 (10–18)
Tidal volume (mL/kg PBW)	6.2 (2.5)
Minute volume (L/min) (median, IQR)	8.3 (6.2–11.0)
FiO_2_ (median, IQR)	0.60 (0.50–0.80)

### Distribution of diaphragm loading conditions during mechanical ventilation

**Fig. 1:**
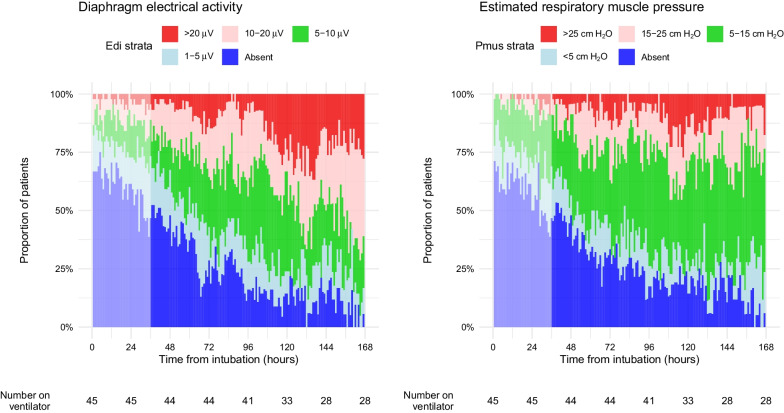
Distribution of diaphragm activity levels and respiratory effort levels during the first week of mechanical ventilation. Left panel: categories of mean hourly diaphragm electrical activity (Edi). Right panel: categories of estimated respiratory muscle pressure (Pmus). Pmus was estimated from the product of hourly diaphragm electrical activity and the daily measurement of neuromuscular coupling

Edi and estimated Pmus varied substantially among patients and over time (Fig. 1). The observed prevalence of different levels of diaphragm activity and respiratory effort is reported in Additional file 1: Table E2. Edi was low or absent (≤ 5 µV) in 51% of study hours (median 71 h per patient, interquartile range 39–101 h). Edi was elevated (> 20 µV) in 10% of study hours (median 1 h per patient, interquartile range 0–17 h).

Similar results were obtained with time series spline extrapolation (Additional file 1: Figure E3). The evolution of diaphragm activity over time was similar between patients intubated for acute lung injury in comparison with those intubated for other reasons (Additional file 1: Figure E4).

### Burden of exposure to dyssynchrony and post-inspiratory loading during mechanical ventilation

**Fig. 2:**
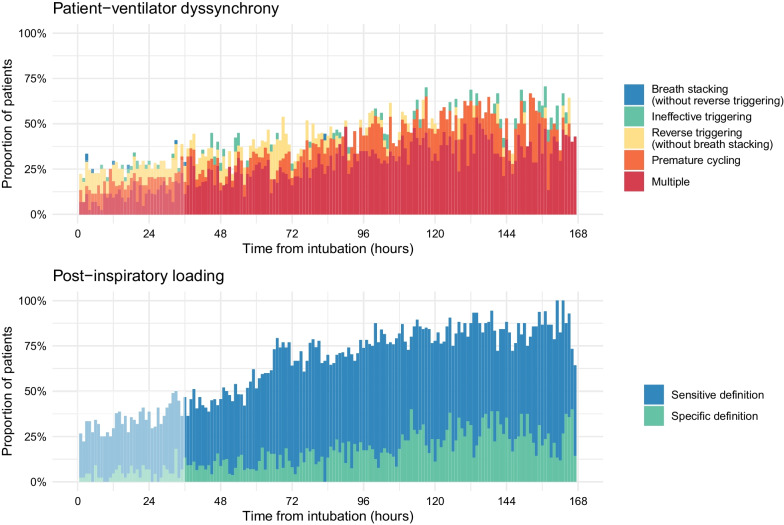
Burden of exposure to patient-ventilator dyssynchrony and post-inspiratory diaphragm contractile loading over time

**Fig. 3:**
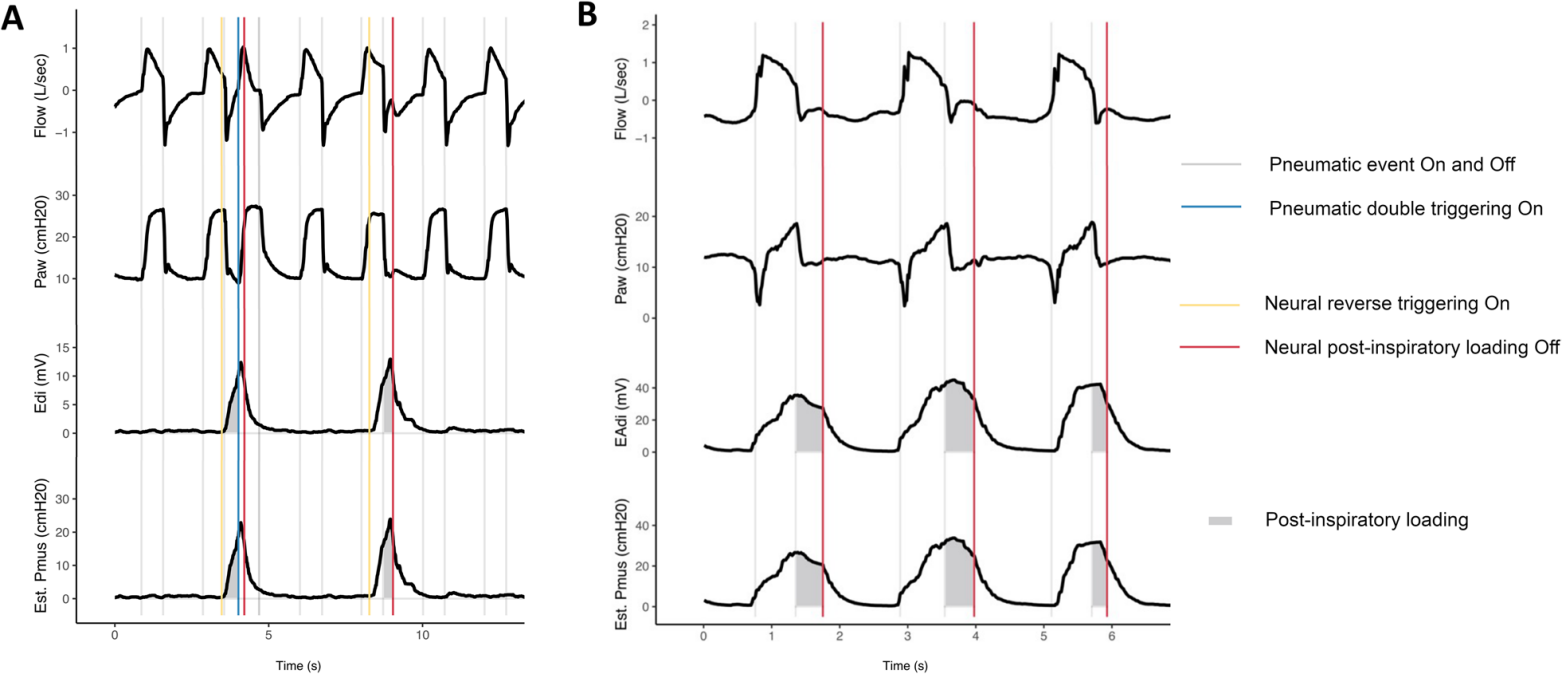
Post-inspiratory loading during reverse triggering and premature cycling. **A** Post-inspiratory loading during reverse triggering. Persistent mechanical effort during the “post-inspiratory” period is evident from the flow tracings, which reveal an attenuated expiratory flow signal indicative of continued diaphragmatic contractile effort. The Pmus waveform is estimated from the Edi waveform and measurement of respiratory neuromuscular coupling (see text for details). The area subtended by the Pmus waveform during the post-inspiratory period (ventilator has cycled off, patient persists with inspiratory effort) is taken as the post-inspiratory effort. In this case, the post-inspiratory loading is attributable to reverse triggering of the patient by the ventilator. **B** In these tracings, the ventilator cycles off at approximately the peak of diaphragm electrical activity, more than 200 ms prior to the end of neural inspiration in most breaths (red vertical lines). Evidence of persistent mechanical effort during this “post-inspiratory” period is evident from the flow tracings, which reveal an attenuated expiratory flow signal indicative of continued diaphragmatic contractile effort. In this case, the post-inspiratory loading is attributable to premature cycling of the ventilator

Patient-ventilator dyssynchrony was common during mechanical ventilation and increased with time (Fig. 2). The most common forms of dyssynchrony were premature cycling and reverse triggering (Additional file 1: Figure E5), but patients not infrequently manifested multiple forms of dyssynchrony in the same recordings (Fig. 2). Post-inspiratory loading (restrictive definition) was present in 12.6% of study hours (median 7 h per patient, interquartile range 2–22 h) (Additional file 1: Table E2), and its prevalence increased progressively over time (Fig. 2). In a sensitivity analysis employing a less restrictive (more sensitive) definition of post-inspiratory loading, it was present in 57% of study-hours (Additional file 1: Table E2, Fig. 2). Figure 3 shows representative tracings of post-inspiratory loading during reverse triggering and premature cycling.

Post-inspiratory diaphragm contractile loading was more likely to occur in hourly recordings where dyssynchronies were observed including reverse triggering (OR 15 for presence of post-inspiratory loading during controlled ventilation, 95% CI 8–35), premature cycling (OR 7.9, 95% CI 6.3–10.0), breath stacking without reverse triggering (OR 4.9, 95% CI 3.1–7.8), and ineffective triggering (OR 2.9, 95% CI 2.2–3.8) (Additional file 1: Figure E6). Pressure support mode was associated with higher Edi, higher estimated Pmus, and greater post-inspiratory loading compared to volume control and pressure control (Additional file 1: Figure E7). Post-inspiratory loading was minimal during CPAP mode (Additional file 1: Figure E7). Post-inspiratory loading was correlated with inspiratory effort (Additional file 1: Figure E8).

### Influence of inspiratory and post-inspiratory loading on diaphragm neuromuscular coupling

**Fig. 4:**
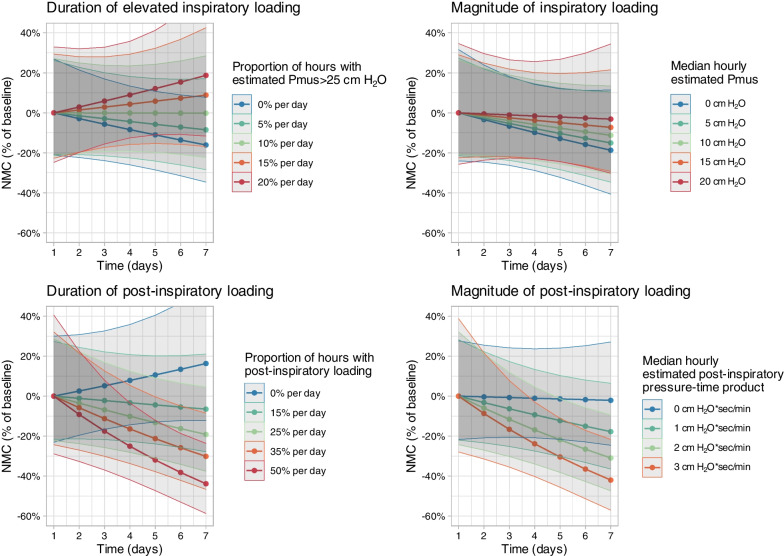
Associations between inspiratory and post-inspiratory loading and changes in diaphragm function assessed by neuromuscular coupling (NMC) over time. Curves represent fitted values computed by linear mixed models examining the interaction between time and exposure to the different loading conditions. Shaded regions represent 95% confidence intervals. The rate of change in diaphragm NMC did not significantly vary with the duration of exposure to elevated inspiratory effort (top left, *p* = 0.076 for interaction testing for differences in slope) or with the magnitude of estimated respiratory muscle effort (top right, *p* = 0.56 for interaction). The rate of decline in diaphragm neuromuscular coupling (NMC) was greater with more prolonged periods of post-inspiratory loading (bottom left panel, *p* = 0.007 for interaction) and with increasing magnitude of post-inspiratory loading (bottom right panel, *p* = 0.009 for interaction)

Neither the duration of elevated inspiratory loading (proportion of hours per day with estimated Pmus > 25 cm H_2_O) nor the magnitude of inspiratory loading (quantified by estimated Pmus) were significantly associated with changes in diaphragm NMC over time (Fig. 4, *p* > 0.05 for interactions, Additional file 1: Table E3). By contrast, both a higher proportion of hours per day with post-inspiratory loading (restrictive definition) and a higher daily median post-inspiratory pressure–time product were associated with a progressive impairment in diaphragm NMC over time (Fig. 4, *p* < 0.01 for interactions, Additional file 1: Table E3). These associations persisted after adjusting for the duration and magnitude of inspiratory loading (Additional file 1: Table E3) and after adjusting for daily severity of organ failure (SOFA score), sepsis, and exposure to neuromuscular blockade (interaction *p* = 0.01). A similar association was also observed using the less restrictive (more sensitive) definition of post-inspiratory loading (*p* = 0.03 for interaction).

Diaphragm thickness tended to increase over time with a greater daily duration of post-inspiratory loading (restrictive definition), but this association did not reach statistical significance (interaction *p* = 0.11, Additional file 1: Figure E9). There was no association between change in diaphragm thickness over time and the duration of elevated inspiratory loading (interaction *p* = 0.50, Additional file 1: Figure E9).

### Diaphragm neuromuscular coupling and diaphragm structure and function

**Fig. 5:**
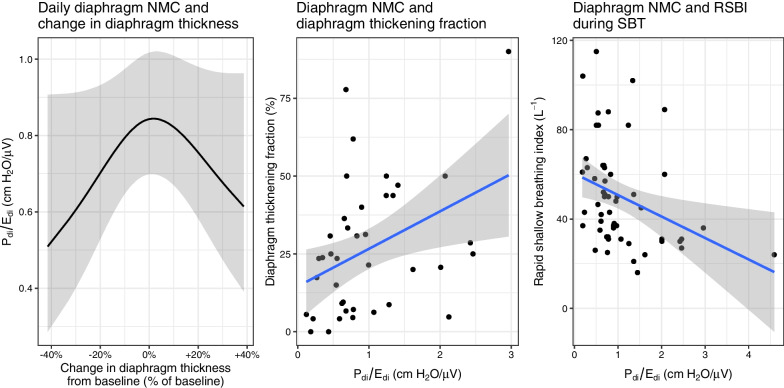
Relationship between diaphragm neuromuscular coupling and markers of diaphragm structure and function. Diaphragm neuromuscular coupling is measured as the ratio of transdiaphragmatic pressure to diaphragm electrical activity, Pdi/Edi). Panel A: both decreases and increases in diaphragm thickness relative to baseline diaphragm thickness were associated with lower neuromuscular coupling (*p* = 0.03, conditional *R*^2^ = 0.54). Panel B: on the final study day, diaphragm neuromuscular coupling was correlated with diaphragm thickening fraction (an ultrasound measure of diaphragm contractility) (*p* = 0.013, *R*^2^ = 0.15). Panel C: diaphragm neuromuscular coupling was correlated with the rapid shallow breathing index (RSBI) measured during a spontaneous breathing trial (*p* = 0.012, *R*^2^ = 0.11)

Both decreases and increases in diaphragm thickness over time were associated with reduced diaphragm NMC (Fig. 5, *p* = 0.03). Lower diaphragm NMC was associated with lower diaphragm thickening fraction on the final day of the study (Fig. 5, *p* = 0.012). Lower diaphragm NMC was also associated with a higher rapid shallow breathing index during spontaneous breathing trials (Fig. 5, *p* = 0.013).

## Discussion

In this study, we found that dyssynchronous post-inspiratory loading of the diaphragm was common during mechanical ventilation and was associated with a progressive impairment in diaphragm function. By contrast, elevated inspiratory loading of the diaphragm was not associated with adverse changes in diaphragm function over time. These observations support the hypothesis that eccentric myotrauma may be an important mechanism of ventilator-induced diaphragm dysfunction in the clinical setting.

Diaphragm disuse leads to rapid muscle atrophy during mechanical ventilation. We found that diaphragm contractility was very low or absent in a majority patients over the first 48–72 h of mechanical ventilation, similar to previous observations by Sklar et al. [24], highlighting the need for timely intervention to maintain diaphragm activity and prevent diaphragm atrophy. Both decreases and increases in diaphragm thickness on ultrasound were associated with impaired diaphragm neuromuscular coupling, suggesting that as previously hypothesized [25] these rapid changes in the diaphragm are indicative of deleterious structural changes in the muscle, possibly including disuse atrophy, inflammation, edema, or replacement fibrosis [26].

In the context of acute systemic illness, the diaphragm may be exquisitely sensitive to injury from excessive loading [9, 10, 27, 28]. We found that elevated inspiratory loading and post-inspiratory loading were common during mechanical ventilation and that various dyssynchronies (especially reverse triggering and premature cycling) increased the probability of post-inspiratory loading. The observation that dyssynchronous post-inspiratory loading—and not (synchronous) inspiratory loading—was associated with impaired diaphragm function suggests that attention to synchrony between patient and ventilator, rather than limiting respiratory effort, should be a priority in order to protect the diaphragm from load-induced injury. Post-inspiratory loading might be attenuated during assisted ventilation by the use of proportional assistance modes (which have better cycling synchrony) or by optimizing the cycling threshold in pressure support. It is possible that post-inspiratory loading may, in some contexts, be of theoretical benefit, as during expiratory braking where the diaphragm acts to maintain lung volume and limit atelectasis during the expiratory phase [3].

### Limitations

This work is subject to a number of limitations. First, owing to the difficulty of enrolling patients in a timely manner and to challenges with artefact in automatically collected waveforms, the number of patients with measurements at any given time is not uniform and the results presented here should be taken as an approximate estimate of the prevalence of these phenomena in patients on the ventilator over time. The validity of the extrapolation to time zero depends on whether data can be assumed to be missing at random. In our judgment there is no systematic factor determining missingness in these physiological data.

Second, respiratory effort was estimated using the technique previously estimated by Bellani et al., where Pmus is calculated as the product of daily NMC and hourly Edi [20]. This approach assumes that NMC is relatively stable over 24 h; variation in NMC over time may introduce some degree of random noise and imprecision in the estimate of hourly Pmus.

Third, we employed neuromuscular coupling (NMC, also termed neuromechanical efficiency) as a surrogate for diaphragm and respiratory muscle function during mechanical ventilation. This measurement may be less sensitive to diaphragm dysfunction in comparison with the gold standard method, transdiaphragmatic pressure during magnetic twitch stimulation of the phrenic nerve. However the twitch magnetic stimulation technique is technically challenging, particularly once patients commence spontaneous breathing, as it requires careful timing to obtain end-expiratory measurements and demonstration of supramaximality and is influenced by coughing and sighing (twitch potentiation) [29]. NMC has been employed as an outcome in multiple clinical trials targeting diaphragm function [30–34] and to characterize changes in diaphragm function over time [35]. The validity of NMC as a measure of diaphragm function is predicated on a linear relationship between Edi and pressure, as shown in multiple previous studies [20, 35, 36]. In support of its validity as a marker of diaphragm function, we found that diaphragm NMC was correlated with multiple markers of impaired diaphragm structure and function, including changes in diaphragm thickness over time, diaphragm thickening contractility on ultrasound, and rapid shallow breathing index (a measure of a patient’s ability to tolerated stressed respiratory conditions) [37]. Others have reported that higher NMC is associated with weaning success [38]. NMC is affected by two critical physiological factors: end-expiratory lung volume and the velocity of contraction [17, 18]. We sought to standardize the force–velocity relation by measuring NMC under quasi-static conditions with the airway occluded [17]. We could not control for variation in end-expiratory lung volume over time, although we found no evidence of an association between PEEP and NMC. NMC measurements may lack precision in mechanically ventilated patients [39], but this statistical “noise” would be expected to bias measured associations between loading conditions and NMC toward the null.

Fourth, the precise timing of the end of diaphragm activation (transition to neural expiration) is uncertain; we chose a relatively conservative threshold for the onset of neural expiration (70% of peak Edi) to avoid overestimating post-inspiratory loading.

Fifth, this study employed automated signal analysis using dedicated software to detect dyssynchrony. Although manual analysis using visual assessment by experts might be considered the gold standard and more reliable, this was not feasible due to the very large volume of waveforms collected. The signal analysis software was previously validated against manual analysis [21]. Each tracing was manually inspected for signal quality. Signal quality criteria were was not defined formally a priori, but artefacts were obvious (absent signal in most cases of discarded recordings) and easy to recognize. Signal quality assessment was performed independently and in duplicate.

Sixth, the observed associations do not establish causation or the direction of causation. It is plausible that higher effort could result from improved diaphragm function. It seems less plausible that improved diaphragm function would reduce post-inspiratory loading (i.e., reverse causation), but these data cannot finally establish a causal relationship.

Seventh, the study population was selected for a high probability of prolonged mechanical ventilation, potentially limiting generalizability of some findings. Nevertheless given the underlying physiological mechanisms the observed association between dyssynchrony and impaired diaphragm function is likely to be generalizable, although the physiological and clinical impact of dyssynchrony may depend on the duration of exposure.

Eighth, discontinuing the study at 49 patients (before reaching the planned sample size of 60) because of slow enrolment and the onset of the Covid-19 pandemic may have limited statistical power. The statistical analyses presented in this paper were pre-planned and were not conducted prior to the decision to stop the study.

## Conclusion

Dyssynchronous post-inspiratory loading of the diaphragm is common in mechanically ventilated patients and may contribute to ventilator-induced diaphragm dysfunction.

### Supplementary Information

**Additional file 1.** Online Supplement.

## Data Availability

The data that support the findings of this study are available on request from the corresponding author.
