# Supplementary material for: Dyssynchronous diaphragm contractions impair diaphragm function in mechanically ventilated patients
Source: Crit Care. 2024 Apr 2;28:107. doi: 10.1186/s13054-024-04894-3 (PMC10988824; doi:10.1186/s13054-024-04894-3)
Supplement: Supplementary file 1 — Additional file 1. Online Supplement. [file 13054_2024_4894_MOESM1_ESM.docx]

Online Supplement

**Dyssynchronous Diaphragm Contractions Impair Diaphragm Function in Mechanically Ventilated Patients**

Benjamin Coiffard

Jose Dianti

Irene Telias

Laurent J. Brochard

Jenna Wong

Jennifer Beck

Christer Sinderby

Niall D. Ferguson

Ewan C. Goligher

**Supplemental Description of Methods**

*Signal acquisition*

The acquisition system (NeuroVent Inc. Toronto, Canada) was connected to the ventilator via RS232 port to acquire airway flow and pressure and diaphragm electrical activity. Esophageal pressure and gastric pressure were directly acquired by the acquisition system. Transpulmonary pressure and transdiaphragmatic pressure were computed by real-time digital subtraction in the signal acquisition software.

Esophageal catheter position was confirmed by the Baydur maneuver. Gastric balloon position was confirmed by gentle abdominal palpation and absence of cardiac artefact, following esophageal balloon positioning confirmation. Balloons were inflated to the validated volume for the dedicated esophageal catheter (1 cc) prior to each pressure measurement. Esophageal and gastric pressure signals were only retained for analysis from once daily recordings obtained after the validity of the measurements and balloon inflation were manually confirmed. During these recordings, intermittent airway occlusions were applied as described in the main text to measure neuromuscular coupling under quasi-static occlusions, provided respiratory efforts were present.

The acquisition system was set to record acquired signals automatically for 5 minutes of every hour. Esophageal and gastric pressure signals were discarded from these recordings as balloon position and inflation was not verified on an hourly basis. Problems with signal artefact in the diaphragm electrical activity due to changing catheter position or disconnections were addressed in quality appraisal. To minimize signal loss from disconnections (accidental, or for ICU transport), clinical staff were instructed to contact the investigators so that recording could be resumed.

*Appraising signal quality*

A team of investigators (BC and JD) visually inspected every hourly recording independently to assess whether the quality of the signals was degraded by artefact. Artefact was deemed present when the Edi signal was intermittently absent or erratic or there was evidence of penetration of cardiac artefact through the signal filter. Poor quality recordings were discarded and the hourly measurements in these cases were treated as missing data. Each recording was adjudicated independently and in duplicate. Cases of disagreement were resolved by consensus.

*Signal analysis*

Signal analysis was performed using dedicated software that automatically detected pneumatic breaths based on inspiratory flow and neural breaths based on Edi. The mean value of Edi (onset to peak diaphragm electrical activity for each neural inspiratory event) in each hourly recording (5 minutes duration) was taken to be representative of the average level of diaphragm activity for the entire hour. If no neural events were identified, the value was taken to be 0 µV. Neuromuscular coupling was computed from the mean value of the ratio of transdiaphragmatic pressure or airway pressure to Edi during each of the occlusion maneuvers performed daily. Hourly Pmus was estimated as the product of the daily neuromuscular coupling measurement (assumed to be relatively stable over 24 hours) and hourly Edi according to the method of Bellani et al. [1].

*Handling missing data*

To minimize bias in estimating the prevalence of varying loading conditions, we had to address two types of missing data problems. First, some hourly recording data was missing from after the patient was enrolled in the study occurred because of failed recordings or poor signal quality with artefact preventing valid measurements. We could conceive of no reason why missingness would be correlated to diaphragm loading condition, and therefore deemed these data to be missing completely at random. To address this missing data problem, we used time series imputation by exponentially weighted moving average to impute missing values in each patient’s hourly time series data, provided there were not more than 5 measurements missing in sequence. Pmus and dyssynchrony rates were taken to be 0 on all hours where Edi was absent, even if neuromuscular coupling measurements were missing or unavailable.

The second missing data problem is more challenging as it pertains to data missing because of delays between intubation and enrolment. To quantify the exact prevalence of diaphragm inactivity, ideally one would commence monitoring and recording diaphragm activity immediately after intubation but this was not feasible for logistical reasons. Given that the magnitude of diaphragm loading is likely to vary over time, especially during the first 3-4 days of mechanical ventilation, time-dependent missingness from delays in enrolment presents an important source of bias in estimates of the prevalence of diaphragm loading conditions (i.e. missingness could systematically bias estimates of the duration of diaphragm inactivity or dyssynchronies, etc.). In this case we deemed data to be missing at random, given the time-dependent nature of the potential bias.

To overcome this problem, we employed model-based extrapolation technique to predict values for diaphragm activity and dyssynchrony in each of the hours prior to enrolment. To assess the sensitivity of the estimates to different extrapolation methods, we employed two promising approaches. First, we fit a Bayesian mixed effects cumulative logistic regression model specifying subject-specific random intercepts and slopes to estimate the probability of being in any of the specific Edi strata over time. In this model, the single fixed effect was time. To ensure that the model reflected the early variation in diaphragm loading conditions, the model was fit on available data from the first 72 hours of mechanical ventilation. This model borrows information both from each individual patient’s trajectory as well as the behaviour of the whole patient population to extrapolate diaphragm activity at any given point in time.

Second, we applied a spline model to each individual patient’s time series data and extrapolated hourly values back to time 0.

The agreement between these two independent and different approaches was evaluated to assess for the sensitivity of the estimated prevalence to the method of extrapolation.

**Table E1**. Definitions of dyssynchrony for signal analysis

| **Dyssynchrony** | **Definition** |
| --- | --- |
| Reverse triggering (with or without breath stacking) | - Overlap between ventilator and patient inspiratory phases - Breath is not triggered by patient - Onset of mechanical inspiration precedes onset of neural inspiration by at least 100 milliseconds - Ventilator is in controlled mode - ΔEdi ≥3 µV |
| Ineffective triggering | - Neural inspiration without a mechanical event - ΔEdi ≥3 µV |
| Breath stacking (with or without reverse triggering) | - Two mechanical insufflation events within a single neural event - ΔEdi ≥3 µV - Note: reverse triggering and breath stacking may coincide |
| Premature cycling | - Overlap between ventilator and patient inspiratory phases - Breath is triggered by patient - Onset of mechanical expiration occurs at least 100 milliseconds before onset of neural expiration - ΔEdi ≥3 µV |
| Post-inspiratory loading (specific definition) | - Overlap between ventilator and patient inspiratory phases - Onset of mechanical expiratory time precedes onset of neural expiratory time (Edi <70% of peak) by at least 200 milliseconds - Mean expiratory flow is >0.33 L/second - End-inspiratory Edi is ≥5 µV higher than baseline Edi |
| Post-inspiratory loading (sensitive definition) | - Overlap between ventilator and patient inspiratory phases - Onset of mechanical expiratory time precedes onset of neural expiratory time (Edi <70% of peak) by at least 50 milliseconds - End-inspiratory Edi is ≥3 µV higher than baseline Edi - No mean expiratory flow criterion |

Dyssynchronies were classified as being “present” in any given hour if there was at least one dyssynchrony event per minute in the hourly recording.

Rationale and Justification for criteria

- Dyssynchrony definitions were developed by consensus among authors
- Previous authors have employed Edi ≥3 as a minimum level of diaphragm activation defining dyssynchrony [2]
- Criteria specified in order to ensure that the dyssynchronous events were “physiologically relevant” in that they involved meaningful differences in timing between ventilator and patient and likely involved some degree of muscle loading
- Minimum differences in timing between ventilator and patient ensure meaningful degree of dyssynchrony
- Minimum Edi criterion ensures some minimum degree of muscle loading
- Mean expiratory flow criterion is intended to require meaningful muscle lengthening
- The onset of neural expiratory time was taken to occur when Edi dropped to 70% of its peak value; this is the threshold used for expiratory cycling in NAVA mode and has been applied in multiple previous studies [3-5]. Previous work suggests that an earlier cycling criterion (i.e. peak Edi) did not match neural expiratory time as it induced increased double cycling [6]. If anything, the 70% threshold is conservative as in healthy subjects breathing without ventilatory support, the transition from inspiratory flow to expiratory flow may occur below 50% of peak Edi [7]. The use of a conservative (higher) value for % of peak Edi would lead us to slightly underestimate the magnitude of post-inspiratory loading.

**Table E2**. Estimated prevalence of respiratory muscle loading conditions over the first 7 days of mechanical ventilation

| **Respiratory muscle loading condition** | **Cohort prevalence^a^**  **(patient-hours, %)** | **Prevalence within patients^b^**  **(%)**  **(median, IQR)** | **Estimated hours of exposure over first 7 days of ventilation^c^**  **(median, IQR)** |
| --- | --- | --- | --- |
| Low or absent respiratory effort |  |  |  |
| Edi = 0 µV | 1795/5102 (35.2%) | 41% (11%, 54%) | 52 (13, 74) |
| Edi 1-5 µV | 788/5102 (15.4%) | 15% (4%, 25%) | 16 (5, 29) |
| Edi <5 µV | 2583/5102 (50.6%) | 59% (29, 85%) | 71 (39, 101) |
| Estimated Pmus <5 cm H_2_O | 2439/4918 (49.6%) | 57% (27%, 79%) | 62 (31, 99) |
| Intermediate respiratory effort |  |  |  |
| Edi 5-10 µV | 1112/5102 (21.8%) | 16% (5%, 32%) | 24 (7, 41) |
| Edi 10-20 µV | 902/5102 (17.7%) | 12% (2%, 25%) | 13 (2, 43) |
| Estimated Pmus 5-15 cm H_2_O | 1527/4918 (31.0%) | 30% (14%, 51%) | 32 (20, 53) |
| Elevated respiratory effort |  |  |  |
| Edi >20 µV | 505/5102 (9.9%) | 1% (0%, 10%) | 1 (0, 17) |
| Estimated Pmus 15-25 cm H_2_O | 634/4918 (12.9%) | 11% (0%, 27%) | 9 (1, 30) |
| Estimated Pmus >25 cm H_2_O | 318/4918 (6.5%) | 0% (0%, 10%) | 2 (0, 14) |
| Adverse patient-ventilator interactions |  |  |  |
| Post-inspiratory loading  (specific definition) | 609/4817 (12.6%) | 5% (1%, 18%) | 7 (2, 22) |
| Post-inspiratory loading  (sensitive definition) | 2723/4817 (56.5%) | 50% (34%, 87%) | 78 (33, 111) |
| Reverse triggering (with or without breath stacking) | 655/4817 (13.6%) | 11% (3%, 21%) | 15 (5, 26) |
| Breath stacking (without reverse triggering) | 249/4817 (5.2%) | 1% (1%, 5%) | 2 (1, 6) |
| Ineffective triggering | 436/4817 (9.1%) | 5% (2%, 13%) | 7 (2, 16) |
| Premature cycling | 1330/4817 (27.6%) | 21% (4%, 41%) | 28 (6, 65) |
| One or more dyssynchronies^d^ | 1899/4817 (39.4%) | 38% (21%, 56%) | 45 (23, 78) |

Edi = diaphragm electrical activity; Pmus = respiratory muscle pressure; ∆P_L,dyn_=dynamic transpulmonary driving pressure (lung-distending pressure); IQR = interquartile range

^a^Cohort prevalence refers to the number of hourly measurements in which the specified condition is observed, as a proportion of the total number of hourly measurements available after imputation of post-enrolment missing data and model-based extrapolation of missing values over the first 48 hours

^b^The proportion of hours in which the specified condition is observed in each patient

^c^The number of hours in which a patient is estimated to be exposed to each condition, computed as the product of the prevalence in each patient and the number of hours the patient is on the ventilator (maximum 168 hours, or the end of day 7).

^d^One or more of reverse triggering (with or without breath stacking), breath stacking (with or without reverse triggering), ineffective triggering, or premature cycling on any given hour

**Table E3**. Statistical models of inspiratory loading, post-inspiratory loading, and diaphragm neuromuscular coupling

In all models: outcome variable = daily measurement of diaphragm neuromuscular coupling (NMC), computed as P_di_/Edi measured during an airway occlusion maneuver

| **Model question** | **Exposure (model term)** | **Model coefficient*** | **p-value** |
| --- | --- | --- | --- |
| Does the **duration** of elevated inspiratory loading (respiratory muscle effort) modify the rate of change in diaphragm neuromuscular coupling? | Proportion of hours per day with estimated Pmus >25 cm H_2_O | -0.37 | P=0.60 |
|  | Time (days) [average rate of change in diaphragm NMC] | -0.03 | P=0.15 |
|  | **Effect of duration of elevated inspiratory loading**  Interaction term (association of rate of change in diaphragm NMC with proportion of hours per day with post-inspiratory loading) | 0.29 | P=0.076 |
|  |  |  |  |
| Does the **magnitude** of inspiratory loading (estimated respiratory muscle effort) modify the rate of change in diaphragm neuromuscular coupling? | Daily median value of hourly estimated Pmus | 0.017 | P=0.10 |
|  | Time (days) [average rate of change in diaphragm NMC] | -0.03 | P=0.25 |
|  | **Effect of magnitude of inspiratory loading**  Interaction term (association of rate of change in diaphragm NMC with daily median value of hourly estimated Pmus) | 0.001 | P=0.56 |
|  |  |  |  |
| Does the **duration** of post-inspiratory loading modify the rate of change in diaphragm neuromuscular coupling? | Proportion of hours per day that meet specific (stricter) criterion for post-inspiratory loading | 1.000 | P=0.023 |
|  | Time (days) [average rate of change in diaphragm NMC] | 0.024 | P=0.33 |
|  | **Effect of duration of post-inspiratory loading**  Interaction term (association of rate of change in diaphragm NMC with proportion of hours per day with post-inspiratory loading) | -0.24 | **P=0.007** |
|  |  |  |  |
| Does the **magnitude** of post-inspiratory loading (daily median post-inspiratory pressure-time product) modify the rate of change in diaphragm neuromuscular coupling? | Daily median value of hourly estimated post-inspiratory pressure time product | 0.14 | P=0.007 |
|  | Time (days) [average rate of change in diaphragm NMC] | 0.00 | P=0.86 |
|  | **Effect of magnitude of post-inspiratory loading**  Interaction term (association of rate of change in diaphragm NMC with daily median value of hourly estimated post-inspiratory pressure-time product) | -0.029 | **P=0.009** |
|  |  |  |  |
| In a model adjusting for both the **duration** of elevated inspiratory loading and the **duration** of post-inspiratory loading, do both inspiratory and post-inspiratory loading have independent effects on the rate of change in diaphragm NMC? | Proportion of hours per day that meet specific (stricter) criterion for post-inspiratory loading | 1.04 | P=0.012 |
|  | Proportion of hours per day with estimated Pmus >25 cm H_2_O | -0.75 | P=0.27 |
|  | Time (days) [average rate of change in diaphragm NMC] | 0.01 | 0.64 |
|  | **Effect of duration of post-inspiratory loading, adjusted for duration of elevated inspiratory loading**  Interaction term (association of rate of change in diaphragm NMC with proportion of hours per day with post-inspiratory loading) | -0.26 | **P=0.002** |
|  | **Effect of duration of elevated inspiratory loading, adjusted for duration of post-inspiratory loading**  Interaction term (association of rate of change in diaphragm NMC with proportion of hours per day with estimated Pmus >25 cm H_2_O) | 0.36 | **P=0.025** |
|  |  |  |  |
| In a model adjusting for both the **magnitude** of inspiratory loading and the **magnitude** of post-inspiratory loading, do both inspiratory and post-inspiratory loading have independent effects on the rate of change in diaphragm NMC? | Daily median value of hourly estimated post-inspiratory pressure-time product | 0.12 | P=0.019 |
|  | Daily median value of hourly estimated Pmus | 0.01 | P=0.29 |
|  | Time (days) [average rate of change in diaphragm NMC] | -0.03 | P=0.31 |
|  | **Effect of magnitude of post-inspiratory loading, adjusted for magnitude of inspiratory loading**  Interaction term (association of rate of change in diaphragm NMC with daily median value of hourly estimated post-inspiratory pressure-time product) | -0.027 | **P=0.01** |
|  | **Effect of magntidue of inspiratory loading, adjusted for magnitude of post-inspiratory loading**  Interaction term (association of rate of change in diaphragm NMC with daily median value of hourly estimated Pmus) | 0.002 | P=0.36 |

Pmus = respiratory muscle pressure

*A **positive** value for the model interaction term coefficient signifies that higher values are associated with an **increase** in diaphragm neuromuscular coupling over time; a **negative** value for the model interaction term coefficient signifies that higher values are associated with a **decline** in diaphragm neuromuscular coupling over time

**
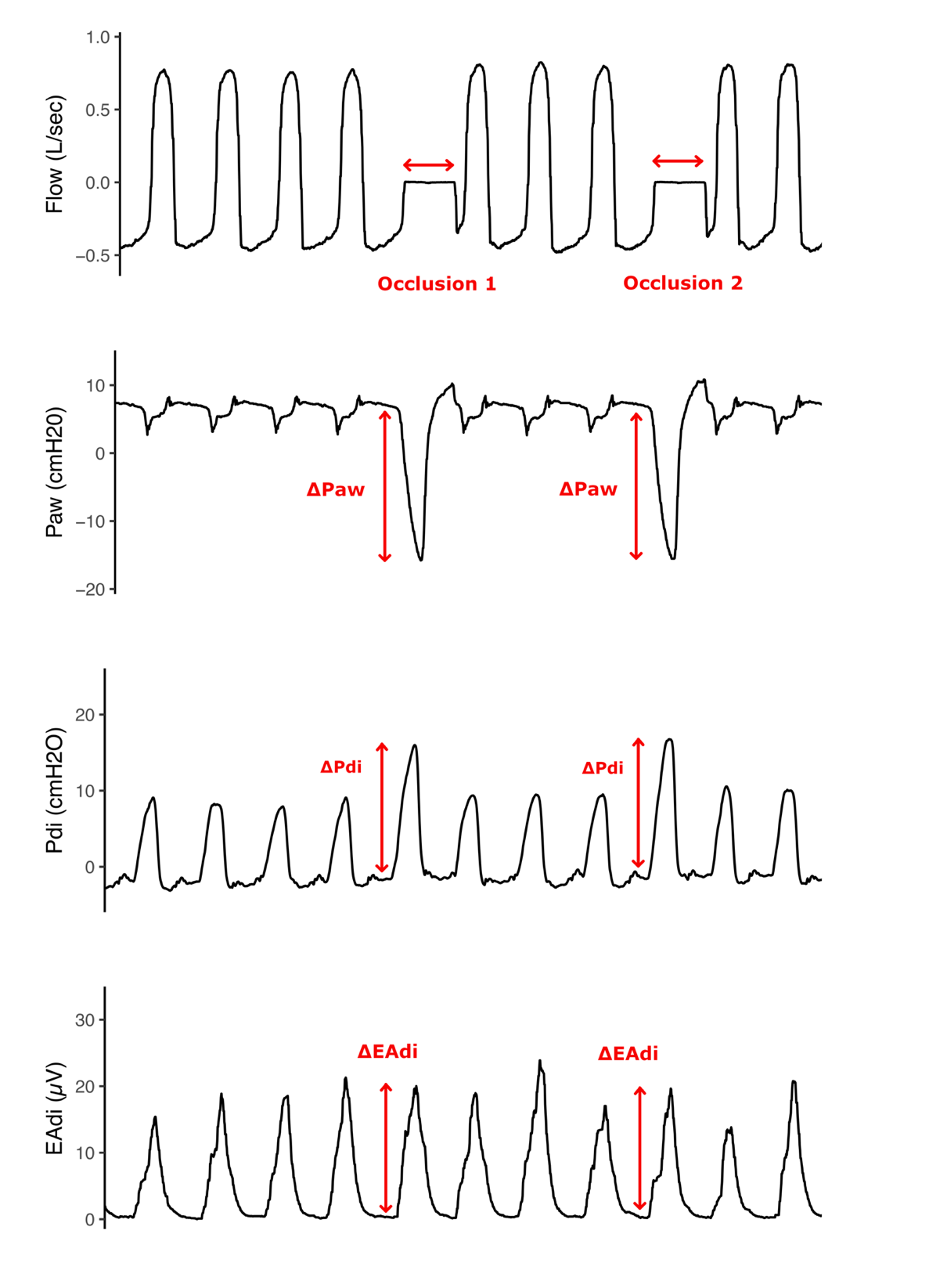
**

**Figure E1.** Representative tracings obtained during the airway occlusion maneuver. Flow, airway pressure (Paw), transdiaphragmatic pressure (Pdi), and diaphragm electrical activity (Edi) were recorded while an airway occlusion was applied at a random interval.

**Figure E2**. CONSORT diagram for study recruitment and enrolment.

**Figure E3**. Prevalence of diaphragm activity over time estimated by extrapolating to time 0 using a spline fit to each individual patient’s time series, performed as a sensitivity analysis to the primary extrapolation method using Bayesian mixed effects cumulative logistic regression.

**Figure E4**. Evolution of diaphragm activity according to whether the primary reason for intubation was acute lung injury. Patients intubated for acute lung injury (acute respiratory distress syndrome or pneumonia) had a broadly similar trajectory of diaphragm activity over time compared to patients intubated for reasons other than acute lung injury (i.e., acute brain injury or non-pulmonary sepsis).


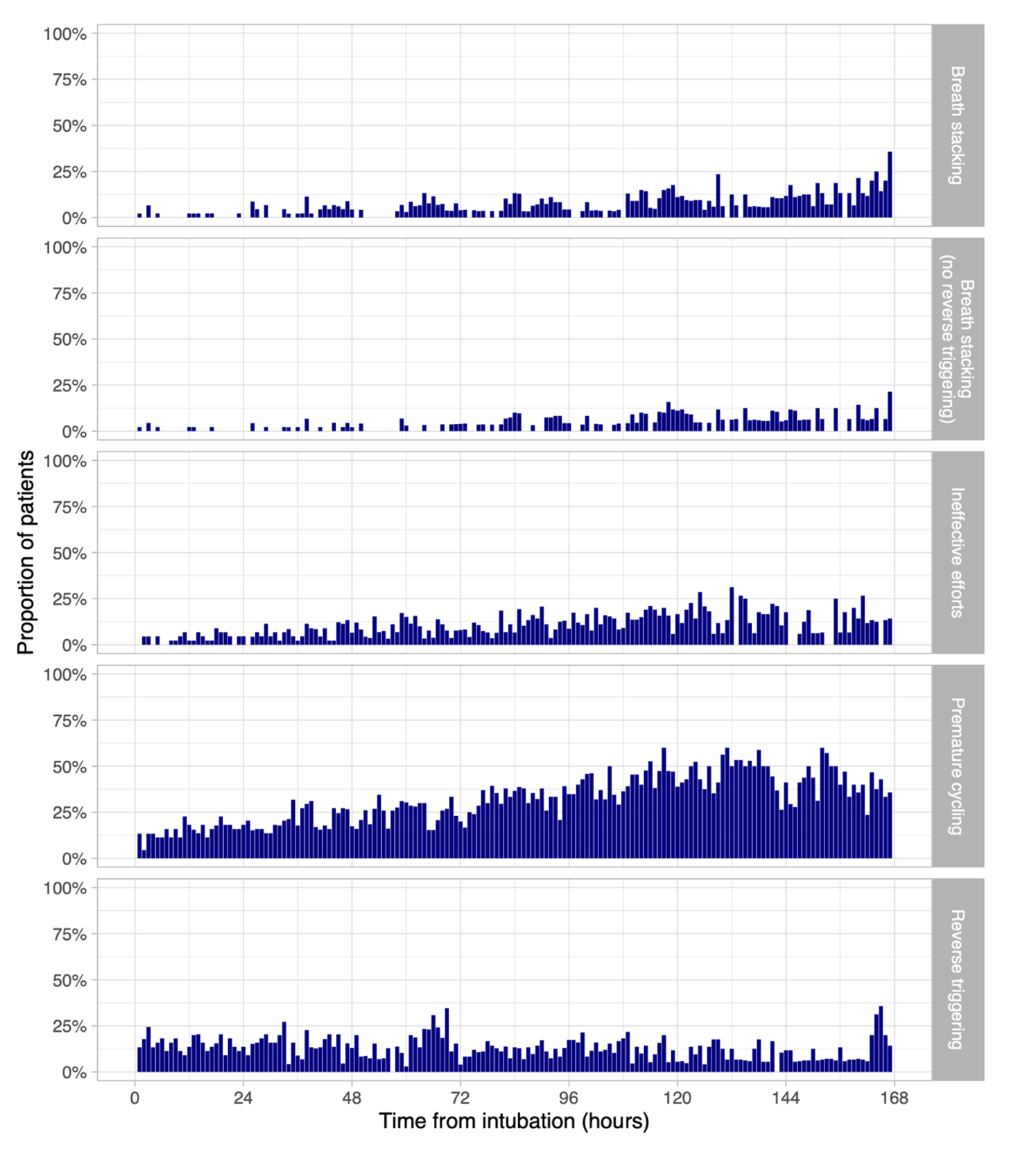


**Figure E5.** Prevalence of patient-ventilator dyssynchrony over time. The top panel shows the prevalence of breath stacking dyssynchrony (with or without reverse triggering); the second panel from the top shows the prevalence of breath stacking dyssynchrony without reverse triggering.


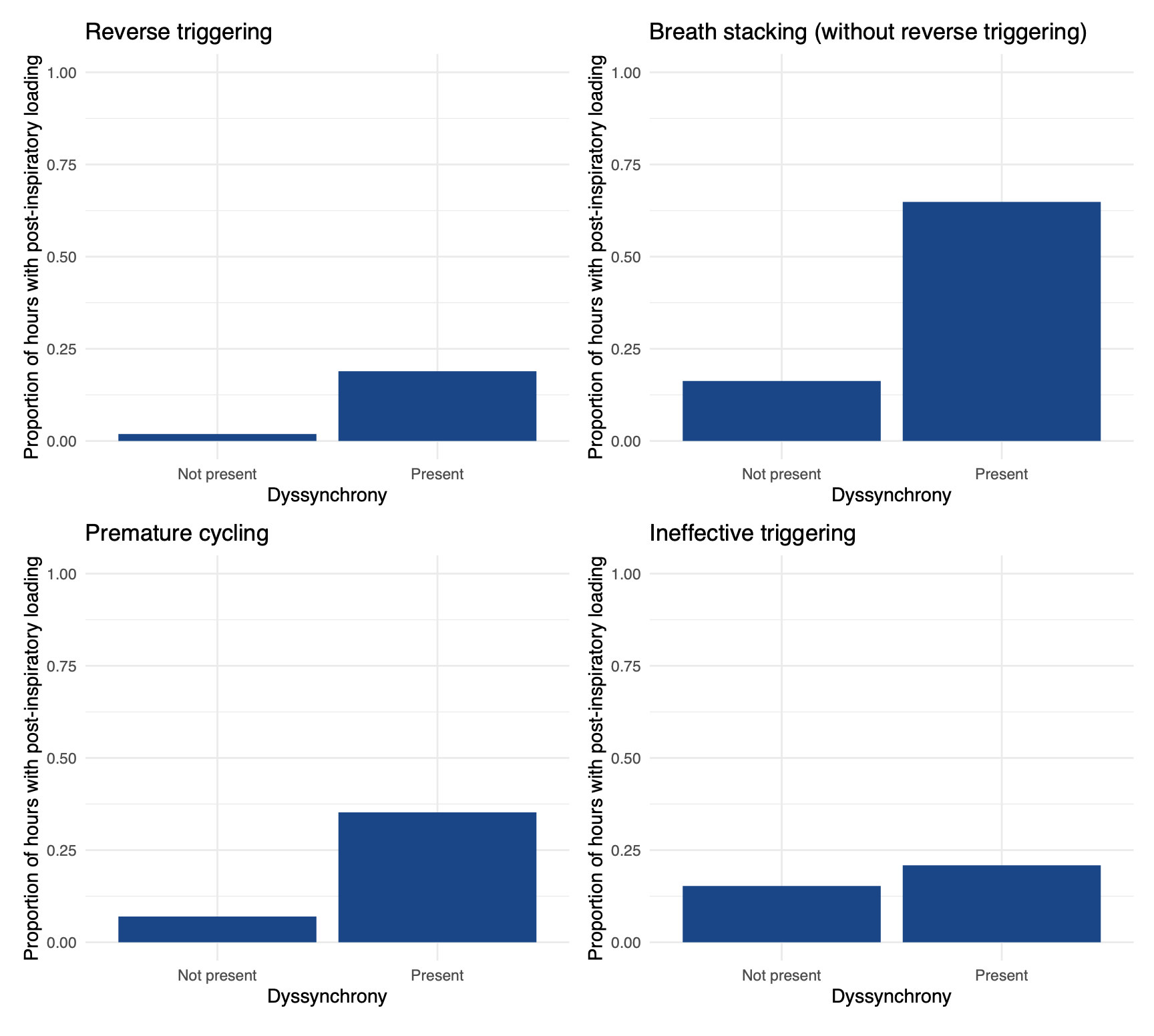


**Figure E6**. Dyssynchronies are associated with an increased prevalence of post-inspiratory loading (defined according to the “specific” definition). Post-inspiratory loading was more likely to occur during controlled ventilation in the presence of reverse triggering (top left, OR 15, 95% CI 8-35), and at any time in the presence of premature cycling (bottom left, OR 7.9, 95% CI 6.3-10.0), breath stacking without reverse triggering (top right, OR 4.9, 95% CI 3.1-7.8), and ineffective triggering (bottom right, OR 2.9, 95% CI 2.2-3.8).

**Figure E7**. Influence of mode of mechanical ventilation on diaphragm loading conditions. The prevalence and magnitude of post-inspiratory loading were higher in pressure support mode and lower in CPAP. After adjusting for differences in the level of inspiratory diaphragm activity, there was no significant difference between pressure support and controlled modes of ventilation.

**Figure E8.** Relationship between inspiratory effort and post-inspiratory loading. Higher hourly mean estimated Pmus per breath was associated with higher hourly mean post-inspiratory loading (p<0.001, marginal R^2^ 0.04, conditional R^2^ 0.24).

**Figure E9**. Relationship between inspiratory effort and post-inspiratory loading and changes in diaphragm thickness over time. Left: There was no significant association between hourly estimated Pmus and changes in thickness of the diaphragm over time (interaction p=0.50). Right: the association between hourly post-inspiratory loading and changes in thickness of the diaphragm over time also did not reach significance (interaction p=0.11).

**References**

1. Bellani G, Mauri T, Coppadoro A et al (2013) Estimation of patient's inspiratory effort from the electrical activity of the diaphragm. Crit Care Med 41:1483-1491. doi: 10.1097/CCM.0b013e31827caba0 [doi]

2. Mellado Artigas R, Damiani LF, Piraino T et al (2021) Reverse Triggering Dyssynchrony 24 h after Initiation of Mechanical Ventilation. Anesthesiology (Philadelphia) 134:760-769. doi: 10.1097/ALN.0000000000003726

3. Liu L, Xu X, Sun Q et al (2020) Neurally Adjusted Ventilatory Assist versus Pressure Support Ventilation in Difficult Weaning: A Randomized Trial. Anesthesiology (Philadelphia) 132:1482-1493. doi: 10.1097/ALN.0000000000003207

4. Liu L, Xia F, Yang Y et al (2015) Neural versus pneumatic control of pressure support in patients with chronic obstructive pulmonary diseases at different levels of positive end expiratory pressure: a physiological study. Critical Care 19:244. doi: 10.1186/s13054-015-0971-0

5. Sinderby C, Liu S, Colombo D, Camarotta G, Slutsky AS, Navalesi P, Beck J (2013) An automated and standardized neural index to quantify patient-ventilator interaction. Critical Care 17:R239. doi: 10.1186/cc13063

6. Piquilloud L, Vignaux L, Bialais E et al (2011) Neurally adjusted ventilatory assist improves patient–ventilator interaction. Intensive Care Med 37:263-271. doi: 10.1007/s00134-010-2052-9

7. Piquilloud L, Beloncle F, Richard JM, Mancebo J, Mercat A, Brochard L (2019) Information conveyed by electrical diaphragmatic activity during unstressed, stressed and assisted spontaneous breathing: a physiological study. Ann Intensive Care 9:89-1. doi: 10.1186/s13613-019-0564-1 [doi]
